# Single Versus Multi-channel Dispersion Analysis of Ultrasonic Guided Waves Propagating in Long Bones

**DOI:** 10.1177/01617346211006660

**Published:** 2021-04-12

**Authors:** Tho N. H. T. Tran, Feng He, Zhenggang Zhang, Mauricio D. Sacchi, Dean Ta, Lawrence H. Le

**Affiliations:** 1 Institute of Biomedical Engineering and Technology, Academy for Engineering and Technology, Fudan University, Shanghai, China; 2 Department of Medical Physics and Biomedical Engineering, University College London, London, UK; 3 Department of Electronic Engineering, Fudan University, Shanghai, China; 4 Department of Physics, University of Alberta, Edmonton, AB, Canada; 5 Department of Radiology and Diagnostic Imaging, University of Alberta, Edmonton, AB, Canada

**Keywords:** ultrasonic guided waves, cortical bone, axial transmission, dispersion, phase/group velocity

## Abstract

Ultrasonic guided wave techniques have been applied to characterize cortical bone for osteoporosis assessment. Compared with the current gold-standard X-ray-based diagnostic methods, ultrasound-based techniques pose some advantages such as compactness, low cost, lack of ionizing radiation, and their ability to detect the mechanical properties of the cortex. Axial transmission technique with a source-receiver offset is employed to acquire the ultrasound data. The dispersion characteristics of the guided waves in bones are normally analyzed in the transformed domains using the dispersion curves. The transformed domain can be time-frequency map using a single channel or wavenumber-frequency (or phase velocity-frequency) map with multi-channels. In terms of acquisition effort, the first method is more cost- and time-effective than the latter. However, it remains unclear whether single-channel dispersion analysis can provide as much quantitative guided-wave information as the multi-channel analysis. The objective of this study is to compare the two methods using numerically simulated and ex vivo data of a simple bovine bone plate and explore their advantages and disadvantages. Both single- and multi-channel signal processing approaches are implemented using sparsity-constrained optimization algorithms to reinforce the focusing power. While the single-channel data acquisition and processing are much faster than those of the multi-channel, modal identification and analysis of the multi-channel data are straightforward and more convincing.

## Introduction

More than 200 million people suffered from osteoporosis-related fractures globally in 2017.^1^ The disease prevalence continues to escalate with worldwide aging of the population over coming decades. The current gold standard of osteoporosis diagnosis is dual-energy X-ray absorptiometry (DXA). However DXA is radiation-based, costly, not portable for bedside application, and does not provide mechanical information of the skeleton. In the last 20 years, quantitative ultrasound (QUS) has been developed to non-invasively characterize the bone tissues due to its potential to reflect both geometrical and elastic properties.^2^ QUS can become a portable and cost-effective diagnostic modality to assess the bone quality for osteoporosis screening and monitoring.

The axial transmission (AT) technique was originally developed to study fracture healing and is currently the most commonly used acquisition configuration to study long bones. In AT setup, ultrasonic transmitters and receivers are placed on the same side along the axial direction of a long bone sample. With the transmitter being held stationary, the receiver is moved at a regular interval to detect the incoming signals. Array transducers can also be used to speed up the data acquisition and to compensate for the overlying soft tissue thickness.^3,4^ At close offsets, strong bulk waves can be observed,^5^ while energetic guided waves (GWs) are more dominant^6^ at far offsets. The time-offset ( t - x ) records thus acquired display fast high-frequency bulk waves and slow dispersive low-frequency GWs. Although the characteristics of bulk waves and GWs are both governed by the elastic properties and the thickness of the cortex, the low-frequency GWs have been found to be more sensitive to the waveguide properties, especially the cortical shell thickness.^2^

Ultrasound GWs propagate in distinct frequency-dependent modes along the cortex.^3,6^ The acquired data are usually transformed into a frequency-based domain to extract the kinematic properties of the GW modes.^2,7^ Two signal processing techniques are normally used to analyze GW data. The first one is the time-frequency ( t - f ) analysis or spectral decomposition. The time series is not stationary, that is, its statistical properties change with time, and thus the frequency distribution is not constant over time. The t - f map displays the temporal variation of spectral characteristics,^8-10^ and is useful to study the dispersion effect of the propagating modes. The transform requires only one time series for each t - f map. The second method is more involved and requires multi-channel or t - x data set. The data is first transformed into the frequency-wavenumber ( f - k ) domain using the 2D fast Fourier transform^11^ or most recently the linear Radon transform.^12,13^ The resulting f - k panel is known as the dispersion map, which can subsequently be transformed into the frequency-phase velocity ( f - $c_{p}$ ) or frequency-group velocity ( f - $c_{g}$ ) map using using $c_{p}=\omega /k$ or $c_{g}=\partial \omega /\partial k$ respectively.

The objective of this study is to investigate the merits and disadvantages of the two methods by comparing them using numerically simulated data and ex vivo data from a bovine bone plate.

## Materials and Methods

### Experiments

#### Bone model

**Figure 1.**
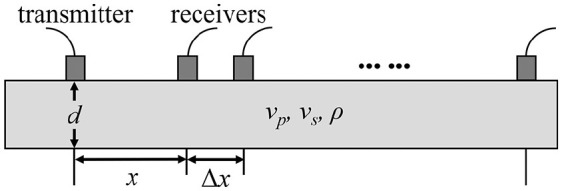
Schematic of AT configuration on a free bone plate.

Figure 1 shows a schematic diagram of AT experimental setup for both numerical and ex vivo measurements. The bone model is a 2D free plate of thickness d, that is, a plate in vacuum. The compressional wave speed, shear wave speed, and cortical density are denoted by $v_{p},$ $v_{s},$ and ρ respectively. The first receiver is placed at an offset x from the transmitter and moved away at a spacing interval Δx.

#### Numerical simulation

The numerical wave fields were simulated by the commercial software package Wave2000 (CyberLogic Inc., New York, NY, USA). The bone plate model used was 5 mm thick with $v_{p},$ $v_{s},$ and ρ being 4000 m/s, 1970 m/s, and 1.9 g/cm^3^, respectively.^5^ A set of 100 time series was computed from 20 to 120 mm offsets at a spatial increment of 1 mm. Each record was 100 μ s long with a time interval of 18.45 ns. Absorbing boundaries were added to both ends of the bone model.

#### Ex vivo data acquisition

The ex vivo measurement was performed on a mid-diaphyseal plate taken from a bovine femur at room temperature of 22°C. The data was acquired by two 1-MHz angle beam compressional wave transducers (Panametrics C548, Waltham, MA, USA) attached to two 30° wedges (Panametrics ABWM-7T-30°). The two broadband transducers had similar peak frequency around 1.06 MHz and a −6 dB bandwidth of 83.5%. Ultrasound gel (Aquasonic 100, Parker Laboratories Inc., USA) was applied as a coupling agent on all contact surfaces. The cortex thickness was 5.8 mm, which was the average value of the thicknesses measured along the bone sample by a digital caliper. $v_{p}$ was 3913 m/s determined by ray tracing while $v_{s}$ = 1900 m/s and ρ = 2.4 g/cm^3^ were taken from literature.^14^ A total of 64 ultrasound records was acquired with 38 mm closest offset, 1 mm spacing interval, 100 μ s time length, and a time interval of 0.1 μ s.

### Signal Processing

#### Single-channel analysis: High-resolution spectral decomposition

The spectral decomposition method described in this section was reported in Bonar.^15^ The recorded time series, s(t) can be considered as a composition of multi-convolution of wavelets, w(t,n) with the corresponding reflectivities, r(t,n),

(1) $$s(t)=\sum_{n=1}^{N}\;w(t,n)*r(t,n)$$

where the wavelet dictionary contains N wavelets. Frequency-varying Ricker wavelet^16^ was used, and the wavelet-dependent reflectivities highlight the change of frequency within s(t), thus giving rise to the local time-frequency representation of the time series. Equation (1) can be written in matrix form:

(2) $$s=\left(\begin{array}{c} W_{1}W_{2}\;\cdots \;W_{N} \end{array}\right)\left(\begin{array}{c} r_{0}\\ r_{1}\\ \vdots \\ r_{N} \end{array}\right)=Wr$$

where W and r represent the wavelet dictionary and the reflectivity sequences.

We seek a high-resolution or sparse solution, **r** by minimizing the following cost function, which has a $l_{2}$ -norm misfit term and a $l_{1}$ -norm constraint,

(3) $$J(r)=∥s-Wr{∥}_{2}+\lambda ∥r{∥}_{1}$$

where λ is the trade-off parameter. Equation (3) poses an $l_{1}$ -norm regularized inverse problem. Since a closed form solution of equation (3) does not exist, an iterative solver known as Fast Iterative Shrinkage Thresholding Algorithm (FISTA)^17^ is employed to seek a solution:

(4) $$r_{j+1}=\mathrm{SOFT}\left(h_{j}+\frac{1}{\alpha }W^{H}(s-Wh_{j}),\frac{\lambda }{2\alpha }\right)$$

where the SOFT thresholding operator is defined by

(5) $$\mathrm{SOFT}\left(Ae^{i\theta },\lambda \right)=\{\begin{array}{ll} (A-\lambda )e^{i\theta } & \;if\;A\;>\;\lambda \;\\ 0 & \;if\;A\;\leq \;\lambda \;, \end{array}$$

and α is a constant that must be greater than or equal to the maximum eigenvalue of $W^{H}W$ and the superscript H denotes Hermitian. The clever update $h_{j}$ is given by

(6) $$h_{j}=r_{j}+\left(\frac{\xi_{j}-1}{\xi_{j+1}}\right)(r_{j}-r_{j-1}),$$

and

(7) $$\xi_{j+1}=\frac{1+\sqrt{1+4\xi_{j}^{2}}}{2}.$$

The FISTA solution has fast convergence rate O( $1/j^{2}$ ).^17^

The theoretical f - $c_{g}$ and f - $c_{p}$ dispersion curves are computed by DISPERSE (Imperial College NDT Lab, London, UK).^18^ The f - $c_{g}$ curves are transformed into the t - f curves using $t=x/c_{g}$ where x is the receiving offset.

#### Multi-channel analysis: High-resolution linear Radon transform

Radon transform, introduced by the Austrian mathematician Johann Radon in 1917, is an integral transform along straight lines. The transform considers the ultrasonic wave fields as a superposition of plane waves and stacks the signal amplitudes along linear trajectories. The application of high-resolution Radon transform (HRRT) to image the dispersion energy of GWs propagating in long bones have been discussed in depth in our previous publications.^12,13^ A mathematical description of the method is briefly provided here.

Let $d(t,x_{n})$ be a series of ultrasonic time signals at different offsets $x_{1},$ $x_{2},$ . . ., $x_{N}$ where t is arrival time. The time signals can be written as a superposition of Radon signals:

(8) $$d(t,x_{n})=\sum_{k=1}^{K}\;m(\tau =t-p_{k}x_{n},p_{k}),\;n=1,\ldots ,N$$

where the slowness p is sampled at $p_{1},$ $p_{2},$ . . ., $p_{K},$ and the time intercept τ is the arrival time at zero-offset. Taking the temporal Fourier transform of equation (1) yields

(9) $$D(f,x_{n})=\sum_{k=1}^{K}M(f,p_{k})e^{-i2\pi fp_{k}x_{n}}$$

or, in matrix notation,

(10) D=LM

where $L=\exp(-i2\pi fp_{k}x_{n})$ is the Radon inverse operator and $L^{H}$ is the adjoint Radon forward operator. The adjoint Radon operator has a poor resolving power and therefore does not provide adequate focusing in the dispersion panel.^13^ To improve the imaging resolution, a regularized Radon solution is often sought. Here, we used the Cauchy-norm regularized Radon solution^12,13^

(11) $$M=(L^{H}L+\mu Q{)}^{-1}L^{H}D$$

where μ is the trade-off parameter, Q is a diagonal weighting matrix with elements

(12) $$Q_{ii}=\frac{1}{\left(1+M_{i}^{2}/\sigma^{2}\right)},$$

and $\sigma^{2}$ is the scale factor of the Cauchy distribution.

Equation (11) provides a high-resolution Radon solution and is a non-linear system of equations, which can be solved by the iterative re-weighted least-squares (IRLS) scheme for each frequency.^12,13^ Finally, the f - $c_{p}$ dispersion map is obtained via $c_{p}=1/p$ and linear interpolation.

## Results

**Figure 2.**
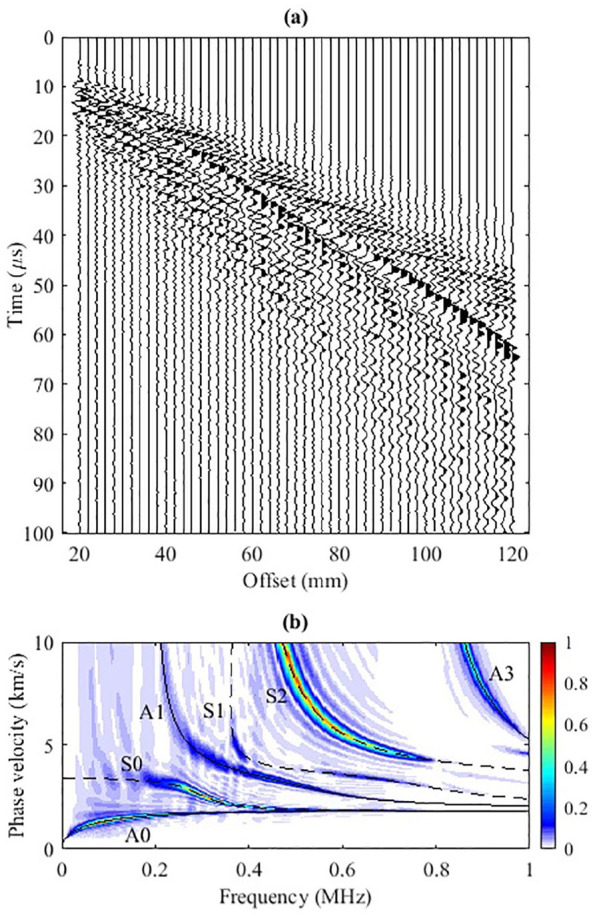
(a) The self-normalized simulated waveforms. (b) The corresponding f - $c_{p}$ dispersion panel. Superimposed in black are the dispersion curves predicted by DISPERSE.

Figure 2 shows the simulated t - x section (Figure 2(a)) and its corresponding Radon f - $c_{p}$ panel (Figure 2(b)). The simulated data was self-normalized and clearly shows the fast high-frequency arrivals and slow low-frequency signals (Figure 2(a)). The Radon panel shows six distinct guided modes, that is, A0, A1, A3, S0, S1, and S2 with the superposition of the theoretical dispersion curves (Figure 2(b)). The energy of these modes spread continuously along a range of frequencies with the energy dominantly distributed within approximately 0-0.2, 0.2-0.4, 0.25-0.55, 0.5-0.8, and 0.86-1.0 MHz for A0, S0, A1, S1, S2, and A3, respectively. Among the six modes, A1 and S1 are weaker. S1 has the discontinuous energy distribution between 0.4 and 0.57 MHz. The energy clusters are well separated and are identified by the dispersion curves, which follow their trajectories.

**Figure 3.**
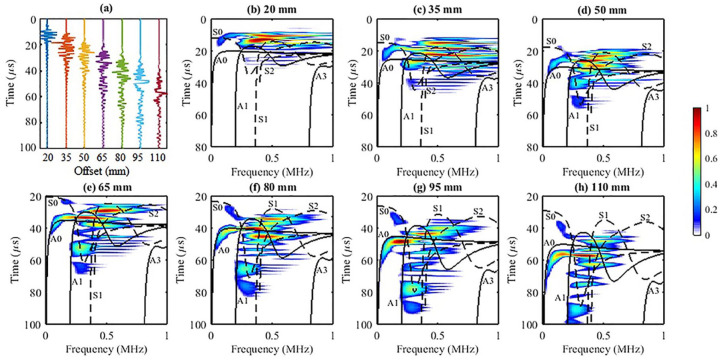
(a) The time series simulated at seven different offsets. (b–h) The corresponding t - f dispersion maps and the superposition of the f - $c_{g}$ curves in black. The black solid and dashed lines in the figures denote the theoretical asymmetric and symmetric modes, respectively. The time axes are shifted upwards for larger offsets while maintaining the same scale as the axes of the near offsets to ensure the visibility of the late-arriving energy.

Seven time series at offsets from 20 to 110 mm with 15 mm spacing are plotted in Figure 3(a). The range of offset covers the near, mid, and far transmitter-receiver distances. The spectral decomposition of the time series is shown in Figures 3(b) to (h). At 20 mm, most of the energy is concentrated within 8 to 25 μ s and spans over all frequencies. Within this range, two strong energy clusters (one thin and one thick) are close to each other but can be identified from approximately 0.3 to 0.75 MHz. Only five modes (A0, A1, S0, S1, and S2) show their presence in this region but none of the dispersion curves intersects the two energetic dispersion clusters. As the offset increases to 35 mm, the whole GW energy spectrum migrates downward and spreads broader along the time axis, displaying dispersion. The two strong energy clusters identified at 20 mm offset are now separated into three clusters bounded between 0.4 and 0.8 MHz. The aforementioned five modes are also present in the energy zone. A0 appears to track the GW energy for all frequencies higher than 0.2 MHz. A3 just touches the rim of the spectrum. As the offset increases from mid offset at 50 mm to far offset, 110 mm, the GW energy travels further, thus shifting the whole spectrum forward in time. There are several observations. First, the energy clusters, which are overlapped at close offset, are well separated with increasing offset and different speeds. Second, the high frequency of the GWs decreases more rapidly than the low frequency not only with time but also with offset as the maximum frequency decreases from 1 MHz at 50 mm to around 0.75 MHz at 110 mm. Thus the whole spectrum becomes high frequency limited. Third, the larger traveling time has mainly low-speed and low-frequency GW energy. Fourth, both methods identify three most energetic modes: A0, S0, and S2. In comparison to the phase velocity Radon map (Figure 2(b)), A3 is basically absent in all these t - f panels.

**Figure 4.**
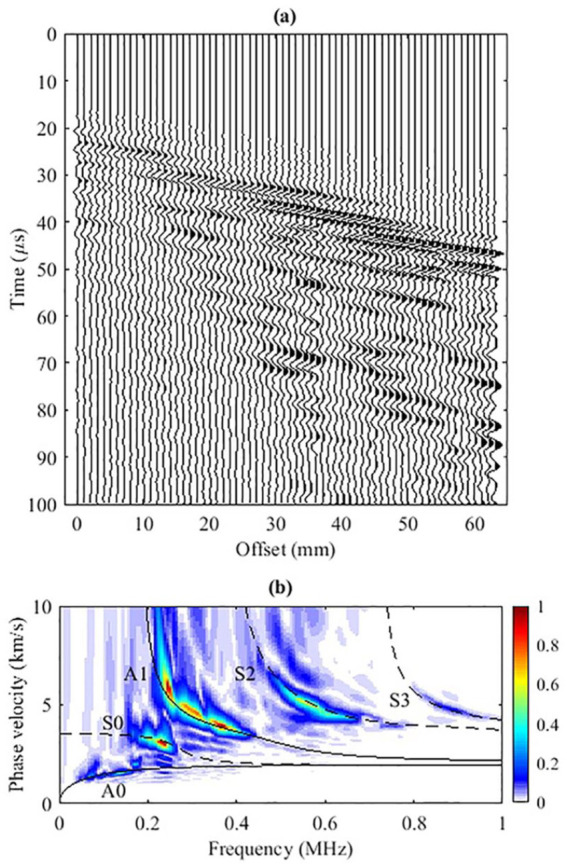
(a) The self-normalized time series versus offset of the ex vivo experimental data. (b) The corresponding f - $c_{p}$ dispersion panel. Superimposed in black are the dispersion curves predicted by DISPERSE.

**Figure 5.**
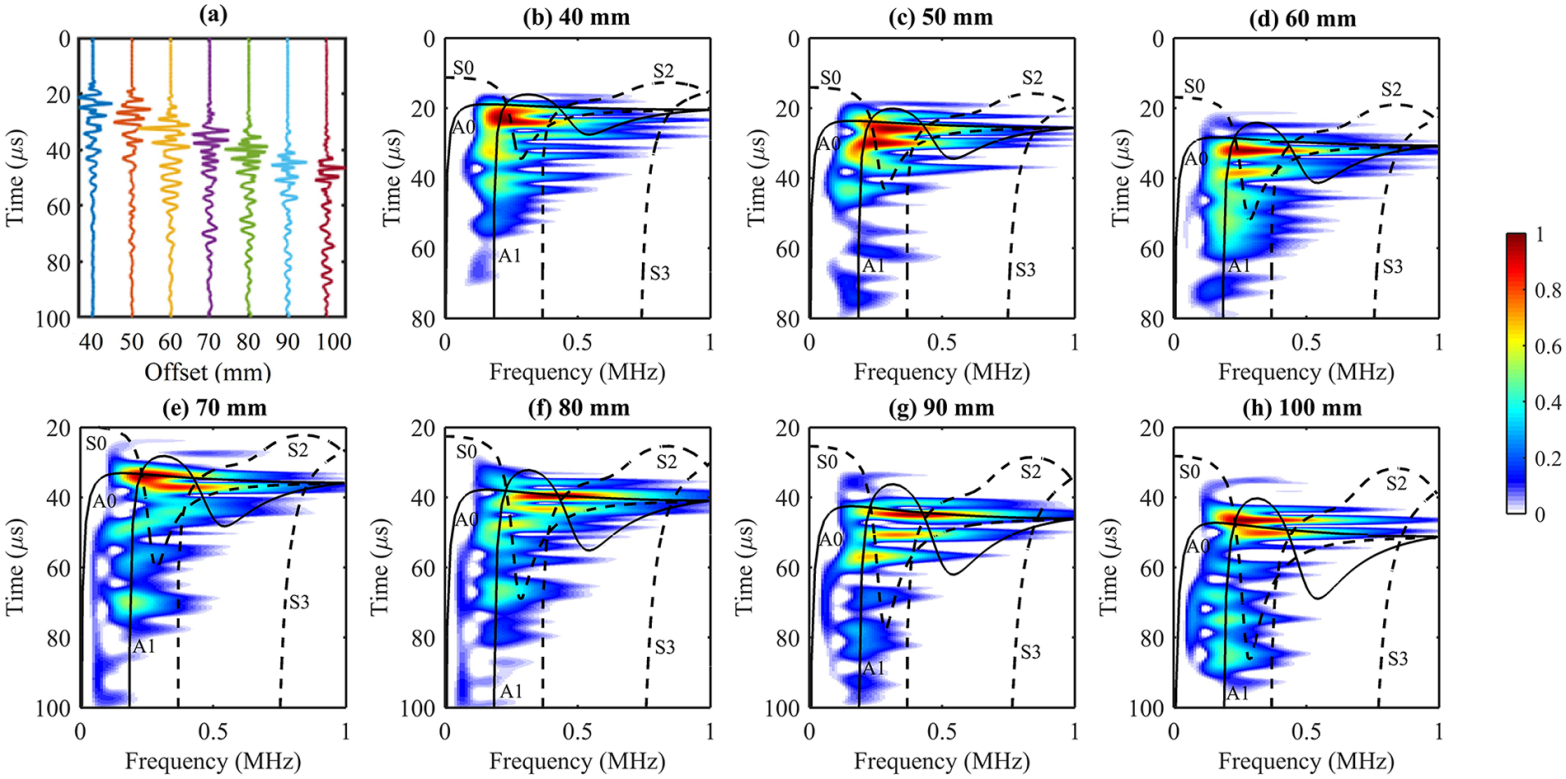
(a) The time series acquired at seven different offsets for the ex vivo experiment. (b–h) The corresponding t - f dispersion maps and the superposition of the f - $c_{g}$ curves in black. The black solid and dashed lines in the figures denote the theoretical asymmetric and symmetric modes, respectively. The time axes are shifted upwards for larger offsets while maintaining the same scale as the axes of the near offsets to ensure the visibility of the late-arriving energy.

Figure 4(a) presents the self-normalized t - x section for the ex vivo data. Unlike the simulated data, the slow-traveling GWs are not obvious. Five GW modes, A0, A1, S0, S2, and S3, can be identified in the Radon panel (Figure 4(b)), and S0, A1, and S2 are the most energetic modes. The energy clusters are confined approximately within 0.05-0.2, 0.18-0.25, 0.25-0.45, 0.45-0.7, and 0.8-1.0 MHz for A0, S0, A1, S2, and S3, respectively. The five modes (A0, S0, A1, S2, and S3) track the GW energy clusters along the trajectories with frequency. Seven time series from 40 to 100 mm with 10 mm spacing are plotted in Figure 5(a). Their spectral decomposition shows the strong energy cluster has high speed but low-to-mid range frequencies (around 0.2–0.6 MHz) around 20 μ s at 40 mm to 45 μ s at 100 mm (Figure 5(b)–(h)). Four modes A0, A1, S0, and S2 show their dominant presence and S3 only crosses the cluster at high frequency. The t - f spectrum decreases from 1 MHz down to not more than 0.5 MHz at around 100 μ s at 100 mm.

## Discussions

The objective of the present study was to compare the single channel and multi-channel methods to analyze GWs at offsets relevant to in vivo data acquisition on long bones. The two most commonly used methods are the time-frequency representation for single channel and 2D f - k or f - $c_{p}$ technique for multi-channel. To facilitate a fair comparison, both methods were posed as a regularized inverse problem to achieve high resolution or sparse solutions.

Fourier transform is the most common method to analyze the frequency content of a time signal by transforming it from the time domain to the frequency domain. By means of Fast Fourier Transform (FFT), the Fourier transform renders a fast and powerful technique to analyze the signals. However one of the drawbacks of Fourier transform is its incapability to provide the time or local attribute of the signal. Information about the variation of frequency content with time can be important and for example, in our case, the change of frequency with time relates to dispersion characteristics of the signal.

Local t - f analysis transforms a 1D time signal into a 2D t - f map, which provides the study of the time-evolution of frequency in the signal. The method only requires one time series for the transform. The existence of a mode is confirmed if its predicted group velocity curve intersects the GW energy clusters. The strong presence of a mode depends on whether the curve intersects the most energetic clusters, which are usually close to the onset of the time signal, and its continuous encounter of the GW energy as time progresses. With increasing travel time, only the low-frequency and slow-speed GWs remain. The method is fast and cost-effective as it only requires one transducer at a fixed position to acquire the time series, and the transform of one record is only performed. However, our data shows the energies of the modes are overlapped especially at close offsets. Even at mid and far offsets, some energy clusters are separated but the trajectories of the modes are not clear in the t - f panel. The dispersion curves cross each other close to the signal onset, and do not conform to any definitive energy trajectories. For multi-channel analysis, the method requires a set of time series acquired at different offsets and thus the processing time takes longer to do the mapping. The GW energy is usually confined in different clusters, which define patterns of trajectories. These trajectories are well separated in the Radon panel, which provides much better resolution than 2D Fourier transform.^13^ The predicted phase-velocity dispersion curves follow the GW trajectories for an extended frequency range and they don’t usually cross. The mode-identification process is much more deterministic and convincing than the t - f technique. With the realization of ultrasound array systems with multi-elements and the CPU computing power, the data acquisition and processing times are not more a concern than the cost of the acquisition system. However, Table 1 summarizes the comparison of single- and multi-channel dispersion analysis based on the experience gained from this study.

**Table 1.** Comparison of Single- and Multi-channel Dispersion Analysis.

	Single channel	Multi-channel
Number of records required	Single	Multiple
Method	Spectral decomposition	2D Fourier or Radon transform
Transform domain	Time–frequency	Frequency–wavenumber
		Frequency–phase velocity
Dispersion curve	Group velocity	Phase velocity
Advantages	Quick data acquisition	Fast data acquisition (array probe)
	Fast data processing	More convincing data interpretation
	Reasonable equipment cost	
Disadvantages	Challenging data interpretation	Time-consuming data acquisition (single transducer)
		Expensive equipment cost (array system)
		Slower data processing

## Conclusions

This paper compared the single-channel and multi-channel techniques using simulated data and ex vivo data of a simple bone plate model. Considering the fact that single-channel analysis has a much simpler measurement setup and can acquire dispersion analysis result faster than multi-channel analysis, single-channel analysis can provide a low-cost and time-efficient application in bone quality assessment. The finding is meaningful for the advance of osteoporosis screening and diagnosis especially in developing countries. In terms of the extracting information from the data, the results have shown that the 2D *f* - *c*_p_ or *f* - *k* analysis provides a more convincing interpretation and mode identification process than the t - f technique, and thus is still a more preferable method. However the model used is simple and the GWs are Lamb waves. The ideal model should consider the bone shape and soft tissues above and below the bone plate. For those models, the dispersion curves might be complicated, and the comparison might be challenging. Further investigation for those complicated bone models is necessary to reach a stronger conclusion.
